# Medical outcomes after canal wall-down mastoidectomy, external ear canal reconstruction, tympanoplasty, and mastoid obliteration for extensive cholesteatoma

**DOI:** 10.1007/s00405-026-10042-0

**Published:** 2026-01-23

**Authors:** Michal Luntz, Keren Hod, Roni Barzilai

**Affiliations:** 1 The Israel Society for Auditory Research, Tel Aviv, Israel; 2 Department of Nutritional Sciences, School of Health Sciences, Ariel University, Ariel, Israel; 3 Assuta Medical Centers, Tel Aviv, Israel; 4 Technion Israel Institute of Technology, The Ruth and Bruce Rappaport Faculty of Medicine, Haifa, Israel; 5 Department of Otolaryngology–Head and Neck Surgery, Rambam Health Care Campus, Haifa, Israel

**Keywords:** Cholesteatoma, Mastoid obliteration, Canal wall-down mastoidectomy, Bioactive glass, Tympanoplasty

## Abstract

**Objective:**

To report medical outcomes and time-related recidivism after extensive cholesteatoma treated with canal wall-down (CWD) mastoidectomy, external ear canal (EEC) reconstruction, tympanoplasty, and S53P4 bioactive-glass mastoid obliteration.

**Methods:**

Retrospective study of 127 ears with extensive cholesteatoma treated at a tertiary center. Patients were scheduled for annual otoscopic and non-echo-planar DW MRI follow-up for up to 4 years. For each postoperative year, only ears attending that visit and all previous yearly visits were analyzed. Main outcomes were: completely epithelialized, dry ear; tympanic membrane and/or posterior–superior EEC retraction; retraction pocket (RP) cholesteatoma; and residual cholesteatoma on MRI.

**Results:**

Follow-up attendance declined from 100% (127/127) in year 1 to 67.2% (41/61) in year 4. A dry, epithelialized ear was achieved in 96.9% (123/127). Retraction developed in 33.9% (43/127), with probabilities of 13.4%, 26.4%, 28.9%, and 34.1% at 1, 2, 3, and 4 years, respectively. RP cholesteatoma occurred in 11.8% (15/127) and residual cholesteatoma on MRI in 7.0% (9/127); 23% of retracted ears progressed to RP cholesteatoma. Children ≤ 11 years had higher rates of retraction and RP cholesteatoma.

**Conclusions:**

Despite counseling on the importance of follow-up, attendance declined over time. CWD mastoidectomy with EEC reconstruction, tympanoplasty, and S53P4 obliteration yielded high long-term dry ear rates in extensive and recurrent cholesteatoma, but complete eradication was not achieved. Younger and re-operated patients were at higher risk of retraction and RP cholesteatoma. Recidivism appeared years after the first surgery, supporting prolonged follow-up. Reporting annual outcome incidence in future studies would enhance understanding of extensive cholesteatoma.

## Introduction

Cholesteatoma is associated with a high infection rate, risk of serious complications, frequent need for multiple surgeries, and hearing loss. It may originate from congenital squamous epithelium clusters (congenital cholesteatoma), but more often results from inadequate aeration of the middle ear (ME) and mastoid air cell system, leading to tympanic membrane retraction and retraction pocket (RP) cholesteatoma. An individual’s tendency toward poor ME aeration may also cause RP cholesteatoma re-development. Another cause of cholesteatoma recidivism is incomplete surgical removal, referred to as residual cholesteatoma. This results from the complex anatomy of the ME cleft and its high density of sensitive structures.

Cholesteatoma surgery is an absolute indication. Repeated surgery is needed for recidivism or uncontrolled infection. The high prevalence and insidious nature of residual cholesteatoma, unless visible via otoscopy, led to the standard practice of “second-look” surgery at 6–12 months after a primary procedure of canal wall-up (CWU) mastoidectomy with tympanoplasty [1]. However, cholesteatoma may reappear years later [2] or even after a second look, showing that this approach alone cannot fully address the risk of missing residual cholesteatoma [3].

A solution to this problem is provided by the breakthrough non-echo-planar diffusion-weighted magnetic resonance imaging (non-EP DW MRI) technique. Non-EP DW MRI can detect cholesteatomas as small as 3 mm, with a sensitivity of 0.94 (CI 0.80–0.98) and specificity of 0.94 (CI 0.85–0.98) [4]. However, hyperintensity on non-EP DWI is not entirely specific for cholesteatoma: lesions with proteinaceous or hemorrhagic content, as well as postoperative granulation or other inflammatory changes, may show similar signal characteristics and occasionally lead to false-positive interpretations. Therefore, non-EP DWI findings should always be correlated with ADC maps, conventional T1- and T2-weighted sequences, and clinical assessment to distinguish cholesteatoma from mimicking lesions and to minimize the risk of false-positive diagnoses. Periodic follow-up MRI during the cholesteatoma recurrence period is expected to increase cumulative sensitivity, approaching 100% [2]. To mitigate the expected associated decline in specificity and the risk of unnecessary revision surgery due to false positives, revision surgery is indicated only for cholesteatoma-suspicious findings that are initially large enough or show growth on follow-up MRI [5]. Beyond its diagnostic value and its role in avoiding unnecessary surgeries, non-EP DWI also carries economic advantages. Cost-effectiveness studies show that MRI-based surveillance can be a cost-effective- and in some cases cost-saving alternative to planned second-look surgery for detecting residual or recurrent cholesteatoma [6, 7].

The surgical approach to cholesteatomas seeks to minimize tissue destruction while ensuring effective removal. Debate continues over the best method for larger cases: CWU mastoidectomy with tympanoplasty or canal wall-down (CWD) mastoidectomy with meatoplasty, with or without tympanoplasty (modified or radical mastoidectomy). Residual cholesteatoma rates are lower with CWD [8]. Meatoplasty aims to enable detection of recidivism if it nevertheless occurs after a CWD procedure, to allow removal in the outpatient clinic in some cases, and to create a long-term self-cleaning cavity [9]. However, aside from being considered non-aesthetic, the meatoplasty opening may initially be too small or narrow over time, limiting adequate cavity cleaning in outpatient settings [10]. Poor CWD cavity management increases the risk of infection due to humidity exposure and promotes debris and keratin accumulation [11], leading to restrictions in water exposure and swimming, significant difficulty with conventional hearing-aid use, the need for lifelong outpatient aural toilet and close follow-up. With non-EP DW MRI available, capable of detecting cholesteatoma when otoscopy cannot, the justification for an open radical (CWD) cavity is increasingly debated. When CWD is needed, it seems reasonable to avoid leaving an open cavity and instead finalize surgery with external ear canal (EEC) reconstruction, mastoid obliteration and tympanoplasty, thereby reducing the disadvantages of a radical cavity. Reconstruction and obliteration aim to recreate a more physiologic, self-cleaning ear canal, permitting more normal daily activities and simplifying long-term outpatient care [12]. Some may object to mastoid obliteration, arguing that it compromises the mastoid’s ability to compensate for negative pressure and may disrupt the ME–mastoid aeration balance. However, mathematical modelling of ME gas pressure regulation, with particular attention to mastoid obliteration, concludes that “mastoid obliteration, as a surgical reduction of the mucosal surface for gas exchange, can improve ME gas pressure balance, resulting in better long-term outcomes” [13].

Various materials can be used for mastoid obliteration [14, 15]. A suitable material should be biocompatible, anti-infectious, non-absorbable, volume-stable, provide a reliable radiological signal, be easily removable if needed, be consistently available, and be cost-effective. Numerous studies have reported good outcomes using S53P4 bio-active glass for obliteration in cases of cholesteatoma [16–21].

Inconsistencies in reporting cholesteatoma surgery outcomes hinder meaningful comparisons between studies [22]. These include variations in patient populations, reported demographic and outcome variables, follow-up durations, and data on cases lost to follow-up [23].

This study reports medical outcomes of extensive cholesteatoma treated with CWD mastoidectomy, EEC reconstruction, tympanoplasty, and mastoid obliteration using annual incidence and data on cases lost to follow-up as the reference framework.

## Materials and methods

### Study design and patients

This is a retrospective review of a prospectively maintained database of all consecutive ears with extensive cholesteatoma treated at a tertiary referral center with CWD mastoidectomy, EEC reconstruction, tympanoplasty, and mastoid obliteration using S53P4 bioactive glass between May 2011 and October 2019. Both primary and revision cases were included, and bilateral cases were recorded as separate ears. Patients with incomplete surgical or follow-up data, or without at least 1 year of postoperative follow-up, were excluded from the analysis. For analysis of a given postoperative year, only those patients who had attended that year’s visit and all previous yearly visits were included. Ears in which residual or recurrent cholesteatoma was diagnosed were included in the analysis only up to the time of diagnosis.

## Main outcome measures and time to achieving/developing the main outcomes

The defined main outcomes were: (i) achieving a completely epithelialized and dry ear; (ii) developing retraction of the tympanic membrane and/or the posterior-superior EEC wall; (iii) identifying RP cholesteatoma by otoscopy; and (iv) detecting residual cholesteatoma on non-EP DW MRI. The time taken to achieve or develop a main outcome was recorded in days.

To explore age-related differences in outcome, we performed an additional analysis stratified by age at surgery into “younger” and “older” groups. The age threshold for this stratification was determined in a data-driven manner and is specified in the Results section, where the corresponding outcome patterns are presented. For each postoperative year, among ears that had attended that year’s visit and all prior annual visits, we calculated for each age group the proportion of ears meeting each main outcome measure.

## Surgical indications, follow-up, and imaging

The decision to perform a CWD procedure was based on disease extent, mastoid pneumatization, and anticipated anatomic challenges, as determined by preoperative otoscopy, high-resolution temporal bone CT, previous operative reports, and intraoperative findings. Indications included a high-riding jugular bulb, anteriorly positioned sigmoid sinus, low tegmen, deep sinus tympani, dehiscence of sensitive structures (e.g., facial nerve canal, lateral semicircular canal, tegmen, sigmoid sinus, dura), and a narrow EEC. Multiple prior surgeries on the same ear also supported the choice of CWD.

If, at follow-up, otoscopy revealed an infected or humid tympanic membrane or EEC, even if only mildly retracted, the canal was filled with Dermacombin ointment (Nystatin 100,000 U; Neomycin 2.50 mg; Gramicidin 0.25 mg; Triamcinolone acetonide 1.00 mg) or chloramphenicol 3% ointment. In the presence of infection, a swab was obtained for culture and a 10-day course of oral ciprofloxacin was prescribed. These patients were then scheduled for an additional “off-schedule” visit within 2–4 weeks to confirm stabilization. Typically, 1–4 such treatment cycles were required for the ear to stabilize. Ears that remained infected despite this predefined course of local and systemic treatment and close follow-up were re-operated since this situation conceptually represents the re-creation of RP cholesteatoma [24]. The persistent instability of these ears also represents a failure to achieve one of the primary surgical goals of a safe, self-cleaning ear. In the context of extensive and repeated cholesteatoma, this seems to justify a low threshold for defining the condition clinically as recurrent cholesteatoma and hence as an indication for reoperation.

At each preoperative and postoperative visit, patients (and parents, when applicable) were counseled regarding the nature of cholesteatoma and the importance of long-term follow-up. Routine follow-up was scheduled at 6 weeks, 3 months, 6 months, and 1 year after surgery, and annually thereafter. At each annual visit, patients underwent otoscopy and non-EP DWI MRI.

Brain MRI was acquired using 1.5-T and 3-T scanners. The standard protocol included axial and coronal T1-weighted, T2-weighted, T2-weighted FIESTA, T2-fluid attenuated inversion recovery (FLAIR), diffusion-weighted imaging (DWI) Non-EPI, with apparent diffusion coefficient (ADC) maps, sagittal T2-weighted sequences, and T1-weighted after contrast administration. Slice thickness ranged from 1 to 4 mm, and sequence parameters (repetition time, echo time) were optimized per scanner to ensure diagnostic image quality.

## Surgical technique

**Fig. 1:**
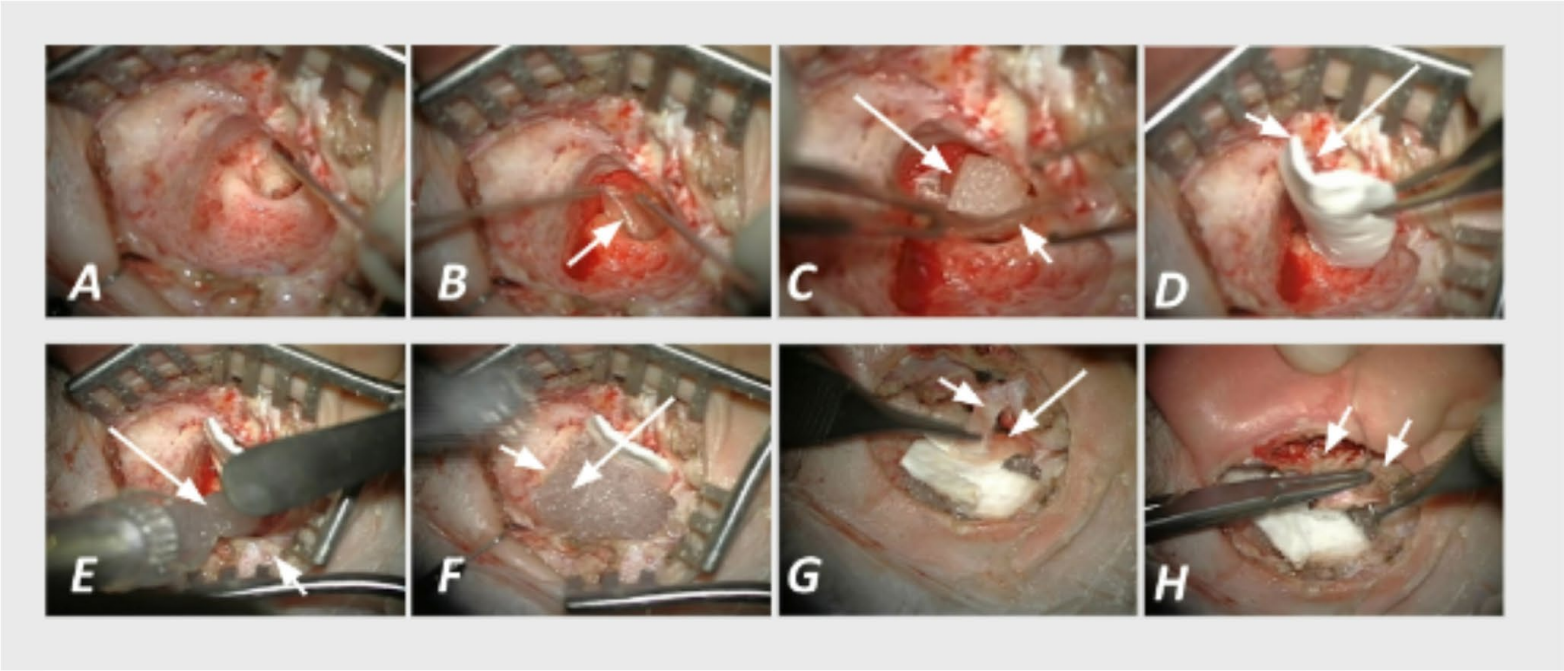
CWD mastoidectomy with reconstruction of the EEC, tympanoplasty and mastoid obliteration using S53P4 BAG. **A**: Formal CWD mastoidectomy procedure; **B**: Tympanoplasty: Placing the tympanoplasty fascia (arrow); **C**: Reconstruction of the EEC: A rectangular piece of periosteum is placed to mark the future location of the posterior-superior wall of the EEC (short arrow). The tympanoplasty fascia (long arrow) is visible beneath the Gelfoam; **D**: Reconstruction of the EEC: A rectangular piece of DuraGen^®^ is positioned behind the periosteum for support (short arrow). The periosteal flap is visible anterior to it (long arrow); **E**,** F**: Mastoid obliteration: The mastoid cavity is filled with S53P4 BAG (arrow); **G**: Closure: The posterior EEC wall skin flap (laterally based) developed at the beginning of surgery (short arrow) is identified and positioned anterior to the periosteum (long arrow). This flap forms the anterior layer of the reconstructed posterior-superior EEC wall.; **H**: Closure: The anteriorly based periosteal flap developed at the beginning of surgery is sutured to become the first layer of ear closure (two arrows). Please note that: (1) The reconstruction of the EEC eventually consists of three layers. The most posterior layer is made of DuraGen^®^, the middle layer is made of periosteal graft, and the foremost layer consists of the laterally based posterior EEC wall skin flap developed at the beginning of surgery. (2) The laterally based posterior EEC wall skin flap developed at the beginning of surgery is quite narrow in this figure (1G, arrow). This is because the EEC of the 4-year-old patient, whose surgery pictures are used for this figure, was very narrow. EEC, external ear canal; CWD, canal wall down; BAG, bioactive glass

The procedure included a traditional CWD mastoidectomy [25] (Fig. 1A), tympanoplasty (Fig. 1B), and posterior-superior EEC wall reconstruction using periosteum (Fig. 1C) supported posteriorly and superiorly by a collagen dural substitute (DuraGen^®^, Integra LifeSciences, Plainsboro, NJ, USA), a clinically established, easy-to-handle collagen matrix (Fig. 1D). The mastoid cavity behind the reconstructed wall was filled with S53P4 bio-active glass (Bonalive^®^, Bonalive Biomaterials, Turku, Finland) (Fig. 1E and F). S53P4 is a non-resorbable, osteoconductive glass with antibacterial properties and a characteristic signal on CT and MRI. It is considered an effective material for long-term middle-ear–mastoid obliteration stability [21]. The granules were packed into the mastoid cavity behind the reconstructed posterior-superior canal wall until the space was filled, typically using approximately 3–9 mL per ear, depending on mastoid size. The laterally based posterior EEC wall skin flap, developed at the beginning of surgery, was placed anteriorly to the periosteum (Fig. 1G). Finally, the anteriorly based periosteal flap, also developed at the beginning, was sutured as the first closure layer (Fig. 1H).

### Statistical analysis

Categorical variables (gender, operated ear side, history of previous surgeries on the target or contralateral ear, contralateral chronic ME disease, follow-up visit attendance, and study outcomes) were presented as frequencies. One-sample Kolmogorov–Smirnov testing indicated non-normal distributions for all continuous variables (age at surgery, number of previous surgeries, follow-up length, and times to epithelialization, retraction, RP cholesteatoma, and residual cholesteatoma). Results were presented as medians and interquartile ranges (IQRs).

Relationships between patients’ characteristics and the main outcomes, between the main outcomes themselves and between the patients’ characteristics themselves, were analyzed using Mann-Whitney and Chi-square tests, as appropriate. Kaplan-Meier survival analysis was used to estimate the cumulative probability of remaining free from RP cholesteatoma and from residual cholesteatoma over the 4-year follow-up period, with time calculated from surgery to the first diagnosis of each endpoint or censoring at last follow-up. Further multivariate analyses were planned to be performed on the basis of findings of the analyses described thus far.

A two-tailed significance test (*p* < 0.05) was used for all analyses. Statistical analyses were conducted with SPSS (Version 28, SPSS Inc., Chicago, IL).

### Artificial intelligence (AI) authoring tools

ChatGPT was used during the writing process to improve the clarity of specific sentences when needed. A native English-speaking professional scientific editor conducted the final language review.

## IRB approval

The study was conducted in accordance with the ethical standards of the Declaration of Helsinki. The study was approved by the Assuta Health Care Institutional Review Board (0004-19-ASMC), with informed consent waived owing to the retrospective nature of the study.

## Results

### Study population and attendance at scheduled yearly follow-up visits (Table 1)

**Table 1:** Consistent successive attendance to the 1st, 2nd, 3rd, and 4th year FU visits (n/N)

FU visit	Number of patients who attended the scheduled visit for a particular year, as well as all the yearly visits leading up to that specific one (*n*)	Number of patients who could potentially be included in the study at each scheduled yearly visit if only time from surgery was considered (*N*)	Attendance to yearly visits (%)
1st Y FU visit	127	127	100%
2nd Y FU visit	106	125	84.8%
3rd Y FU visit	76	99	76.8%
4th Y FU visit	41	61	67.2%

*FU *follow-up; *n *patients who attended the scheduled visit for a particular year, as well as all the yearly visits leading up to that specific one; *N *patients who could potentially be included in the study at each scheduled yearly visit if only time from surgery was considered; *Y *yearly

This retrospective cohort included 127 ears in 125 patients with extensive cholesteatoma treated at a tertiary referral center with single-stage CWD mastoidectomy, EEC reconstruction, tympanoplasty, and mastoid obliteration using S53P4 bioactive glass between May 2011 and October 2019. The median age at surgery was 19 years (interquartile range 10–36), and the mean age was 24.4 ± 17.7 years; of the 127 ears, 63 (49.6%) belonged to patients younger than 18 years. Surgery was a revision procedure for chronic otitis media (COM) in the same ear in 67/127 ears (52.8%); among these, 20 (29.9%), 39 (58.2%), and 8 (11.9%) ears had undergone 1–3, 4, and 5–8 prior operations, respectively. Contralateral COM was present in 58/127 ears (43.9%), and 27 of these (46.5% of this subgroup; 21.3% of the whole cohort) had undergone surgery on the contralateral ear.

#### Achieving a completely epithelialized and dry state (Table 2)

Of the 127 ears, 123 (96.9%) reached a completely epithelialized and dry state within a median of 96 days (IQR, 60.0-196.0). Some ears experienced intercurrent episodes of ‘instability’ (infection/humid ear) and were treated as described above (Materials and Methods). At the 1st, 2nd, 3rd and 4th yearly visits, 92.1%, 89.6%, 82.9%, and 85.4% of ears, respectively, were completely epithelialized and dry.

**Table 2:** Percentages and number of ears that achieved or developed each of the main outcomes at each of the yearly FU visits and the time taken to achieve/develop them

		The condition achieved/developed (main outcome measure)			
		Completely epithelialized and dry ears	Retraction	RP cholesteatoma*	Residual cholesteatoma**
% and n/N of ears that achieved or developed the condition at the time of:	1st Y FU visit	92.1 (117/127)	13.4 (17/127)	0.8 (1/127)	0.8 (1/127)
	2nd Y FU visit	89.6 (95/106)	26.4 (28/106)	4.7 (5/106)	2.8 (3/106)
	3rd Y FU visit	82.9 (63/76)	28.9 (22/76)	9.2 (7/76)	3.9 (3/76)
	4th Y FU visit	85.4 (35/41)	34.1 (14/41)	4.9 (2/41)	4.9 (2/41)
Time taken to achieve/develop the condition (main outcome measure) [days, median(IQR)]		96.0 (60.0-196.0)	426.0 (315.0-633.0)	777.0 (417.0-1062.0)	757.0 (539.5–1092.0)

*RP *retraction pocket; *n *number of patients who achieved or developed the outcome; *N *number of patients included in the study analysis for each yearly visit (patients who attended all yearly visits leading up to the final data collection), *Y *yearly; *FU *follow up; *IQR *interquartile range

*After a diagnosis of RP cholesteatoma, patients were referred for surgery and removed from the study analysis

**After a diagnosis of residual cholesteatoma, patients were referred for surgery and removed from the study analysis

#### Retraction of the tympanic membrane and/or the posterior-superior EEC (Table 2)

Of the 127 ears, 43 (33.9%) developed retraction during the study period, with a median development time of 426 (315–633) days. The likelihood of an ear to be retracted at 1, 2, 3 and 4 years after surgery was 13.4%, 26.4%, 28.9%, and 34.1%, respectively.

#### RP cholesteatoma (Table 2)

Of the 127 ears, 15 (11.8%) developed RP cholesteatoma during the study period, with a median development time of 777 (417–1062) days. In 14 of the 15 ears that developed RP cholesteatoma, this was detected at the 2nd yearly visit or later. The annual incidence (new cases) of RP cholesteatoma was 0.8% (1/127) during the 1st yearly follow-up visit, 4.7% (5/106) during the 2nd, 9.2% (7/76) during the 3rd, and 4.9% (2/41) during the 4th.

#### Residual cholesteatoma (Table 2)

Annual MRI scans were available for all ears. Of the 127 ears, 9 (7.0%) developed residual cholesteatoma, with a median development time of 757 (539.5–1092) days. All of these ears were dry and well-epithelialized and did not develop retraction during the yearly follow-up visits that preceded the diagnosis of residual cholesteatoma. In 8 of these 9 ears, residual cholesteatoma was detected at the 2nd yearly visit or later. The annual incidence of residual cholesteatoma (new cases) was 0.8% (1/127) during the 1st yearly follow-up visit, 2.8% (3/106) during the 2nd, 2.8% (3/76) during the 3rd, and 4.9% (2/41) during the 4th. 

**Fig. 2:**
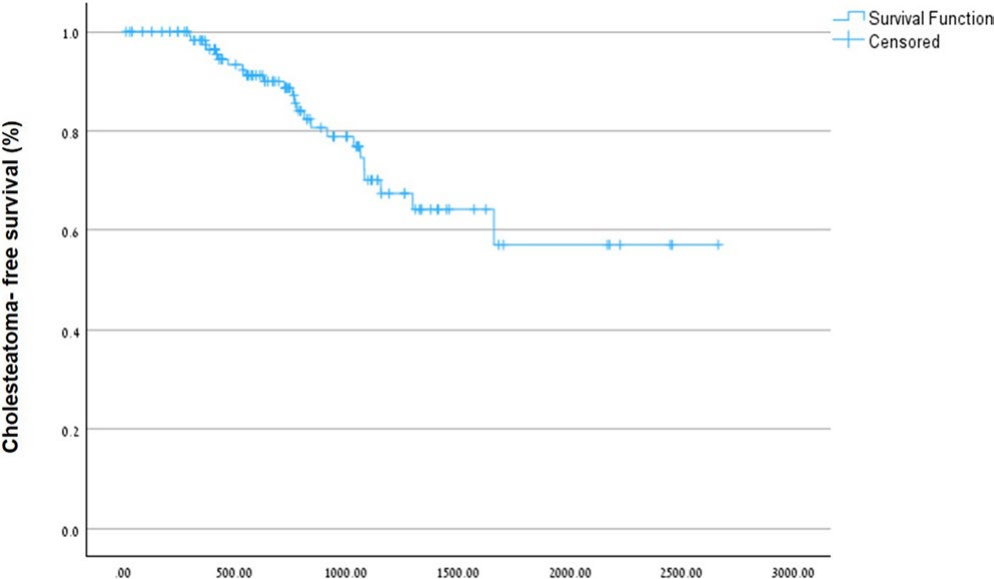
Kaplan–Meier curves showing the cumulative probability of remaining free from retraction pocket (RP) cholesteatoma and from residual cholesteatoma over the 4-year follow-up period after canal wall-down mastoidectomy with external ear canal reconstruction, mastoid obliteration, and tympanoplasty. Time zero is the date of surgery. Censored cases, marked by “+”, indicate ears that remained recurrence-free up to their last follow-up visit but were no longer observed thereafter (loss to follow-up or end of study period). Patients who remained in follow-up without developing RP or residual cholesteatoma are represented within the horizontal segments of the curves

A Kaplan- Meier curve (Fig. 2) illustrates the cumulative probability of an ear remaining free from RP cholesteatoma and from residual cholesteatoma over the 4-year follow-up period.

#### Natural history of retraction and RP cholesteatoma (Table 3)

Of the 43 ears that developed retraction during the study period, 29, 15 and 5 were available for 1, 2, and 3 years of follow-up, respectively, after retraction was first detected. Of those ears, none had regained normal ME aeration 1, 2, or 3 years after that detection. At 1, 2, and 3 years after its detection, 68.9% (20/29), 93.3% (14/15), and 100% (5/5), respectively, remained retracted, while 31.1% (9/29), 6.7% (1/15), and 0% respectively, had developed RP cholesteatoma by 1, 2, and 3 years later. None of the retracted ears developed residual cholesteatoma.

**Table 3:** Natural history of Retraction of TM and/or posterior EEC wall after Retraction was detected for the first time (*N* = 43)

		1y after detection of retraction *N* = 29	2y after detection of retraction *N* = 15	3y after detection of retraction *N* = 5
Condition of the ears	Normal	0	0	0
	Retraction	68.9% (20/29)	93.3% (14/15)	100% (5/5)
	RP chol.	31.1% (9/29)	6.7% (1/15)	0
	Res. chol.	0	0	0

*Y *year; *N *number of ears with the condition; *RP chol*. retraction pocket cholesteatoma; *Res. Chol. *residual cholesteatoma

### Relationship between patients’ characteristics and the main outcomes, between the main outcomes themselves, and between the patients’ characteristics themselves

Using univariate analysis, the following significant relationships were found: Between retraction and RP cholesteatoma (*p* = 0.007), a higher percentage of patients with retraction developed RP cholesteatoma (23.3%) compared to those without retraction (6.0%); between the number of previous surgeries and the development of RP cholesteatoma (*p* = 0.007), a higher percentage of patients who underwent 5 or more surgeries developed RP cholesteatoma (50.0%) compared to those who underwent fewer than 5 (9.2%); between the patients’ age and the development of retraction (*p* < 0.001), patients who developed retraction had a younger median age at surgery (11 years, IQR 8.0–23.0) than those who did not develop retraction (25 years, IQR 12.0–40.8); and between the length of the follow-up period (from the date of surgery to the last visit) and the development of retraction, RP cholesteatoma, and residual cholesteatoma detection, patients who developed these conditions had longer follow-up periods than those who did not: 1,140 days vs. 682 days (*p* < 0.001) for retraction; 1,754 days vs. 736.5 days (*p* < 0.001) for RP cholesteatoma; and 1,177 days vs. 767.5 days (*p* = 0.018) for residual cholesteatoma.

Relationships found to be statistically significant upon univariate analysis (*p* < 0.05) were further analyzed on multivariate analysis, adjusted for age (above or below 11 years), gender, number of previous surgeries (1–4 and 5 or more), and the presence of contralateral ME ear disease. The multivariate analysis revealed significant associations between retraction and RP cholesteatoma development (odds ratio [OR] = 4.674, *p* = 0.012); between the number of previous surgeries and RP cholesteatoma development (OR = 8.116, *p* = 0.010); between patients’ age and retraction development (OR = 0.1750, *p* = 0.002); between follow-up duration and retraction development (OR = 1.001, *p* = 0.003); and between follow-up duration and RP cholesteatoma development (OR = 1.002, *p* < 0.001). Multivariate analysis did not reveal a statistically significant relationship between follow-up duration and residual cholesteatoma development.

### Outcomes stratification by age (Fig. 3 and Appendix)

**Fig. 3:**
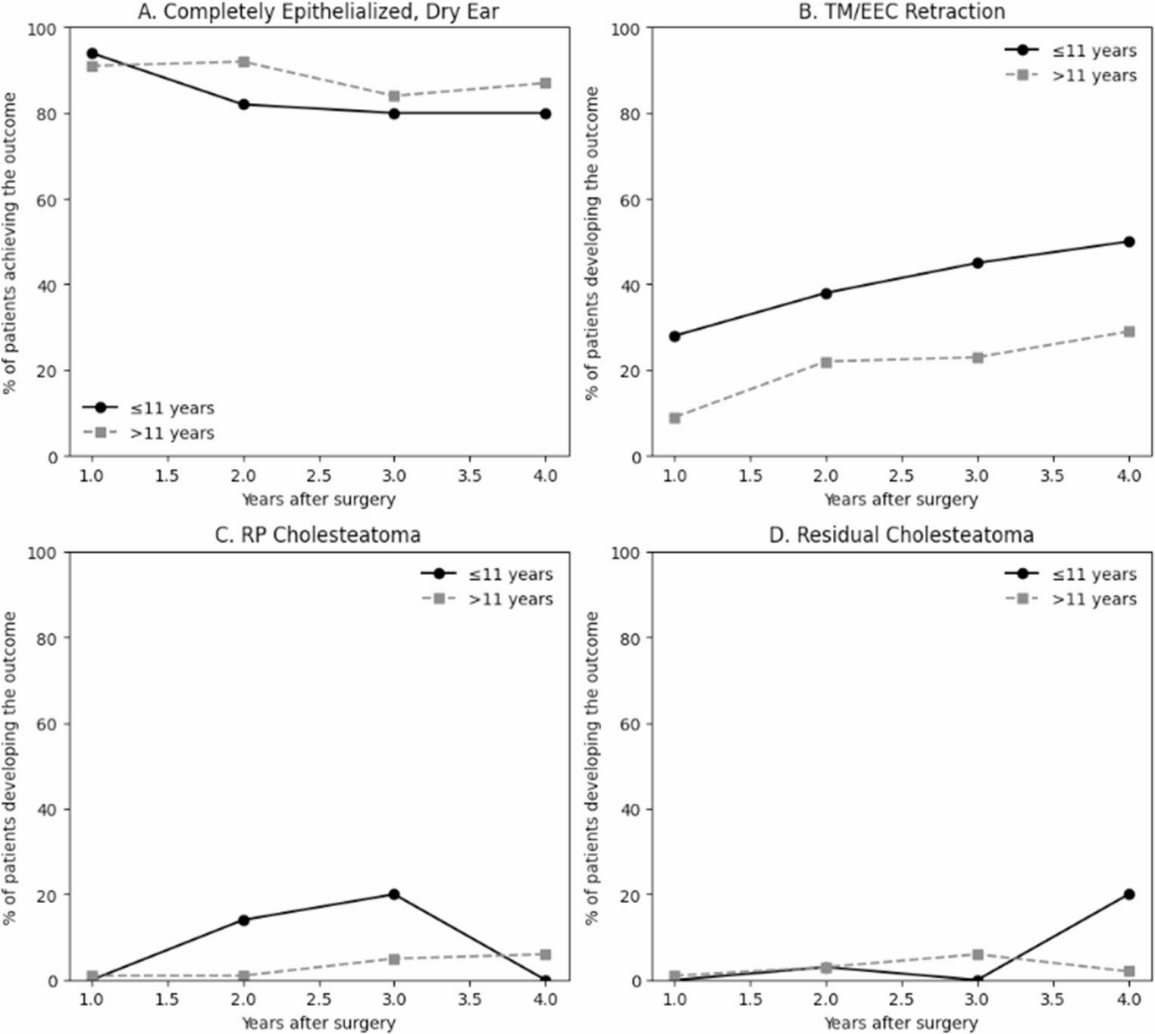
Comparison between children ≤ 11 years and older patients (> 11 years): (**A**) Annual achievement of a completely epithelialized, dry ears (**B**) Annual development of tympanic membrane and/or posterior EEC wall retraction (C) Annual development of RP cholesteatoma (D) Annual detection of residual cholesteatoma on non-EPI DW MRI. Younger children (≤ 11 years) demonstrated higher rates of postoperative retraction and a trend toward higher RP cholesteatoma incidence. EEC; external ear canal, RP; retraction pocket, non-EPI DW; non-echo-planar diffusion weighted imaging, magnetic resonance imaging

Outcomes were stratified by age at surgery into children ≤ 11 years and older patients (> 11 years) (Fig. 3 and Appendix). The proportion achieving a completely epithelialized, dry ear remained high in both groups, with similar rates at 1 year (93.9% vs. 91.5%) and 4 years (80.0% vs. 87.1%; *p* = 0.348), although younger children showed a lower rate at 2 years (82.8% vs. 92.2%; *p* = 0.043). In contrast, tympanic membrane and/or posterior EEC wall retraction were more frequent in children, significantly so at 1 year (27.3% vs. 8.5%; *p* = 0.014), with a persistent difference at later time points. RP cholesteatoma likewise tended to be more common in younger children, with a significantly higher rate at 2 years (13.8% vs. 1.3%; *p* = 0.019). Residual cholesteatoma on non-EP DW MRI did not differ significantly between the two age groups.

## Discussion

Based on structured otoscopic and non-EP DW MRI follow-up, this study reports postoperative outcomes in a unique cohort of extensive and repeatedly operated ears (52.8% had undergone previous surgery. Of those 29.9% had 1–3 prior operations, 58.2% had 4 prior operations, and 11.9% had 5–8 prior operations) that underwent CWD mastoidectomy, EEC reconstruction, tympanoplasty, and mastoid obliteration using S53P4 bioactive glass. Despite repeated counseling about the importance of long-term surveillance, patients’ attendance at postoperative follow-up visits declined from 100% in the first postoperative year to 67.2% by the fourth year (Table 1). This decline, together with the ongoing risk of recurrent infection, retraction, RP cholesteatoma, and residual cholesteatoma if left untreated, illustrates the practical difficulty of maintaining adequate long-term follow-up in this patient population. These findings emphasize the need to develop more robust follow-up strategies and shared-care models to ensure early detection and management of recidivism.

With the surgical technique used in the present study, the likelihood (all respectively) of an ear remaining dry at 1, 2, 3, and 4 years postoperatively was 92.1%, 89.6%, 82.9%, and 85.4%, while the likelihood of its retraction was 13.4%, 26.4%, 28.9%, and 34.1%. The annual incidence (new cases) of RP cholesteatoma was 0.8% (1/127), 4.7% (5/106), 9.2% (7/76), and 4.9% (2/41), while residual cholesteatoma incidence was 0.8%, 2.8%, 3.9%, and 4.9% (Table 2).

Complete cholesteatoma eradication through surgery alone remains unattainable. Evidence-based, effective, and affordable solutions are needed to eliminate small matrix nests and address the longstanding challenge of ME aeration, a known barrier to ME-mastoid health. Among ears with retraction, none regained normal aeration, and 10 (23%) progressed to RP cholesteatoma (Table 3). A significant association was found between retraction and RP cholesteatoma (OR = 4.674, *p* = 0.012), with 23.3% of retracted ears developing RP cholesteatoma compared to 6.0% of non-retracted ears. By one year after retraction, 31.1% (9/29) had developed RP cholesteatoma.

This study identifies a risk group for RP cholesteatoma after CWD mastoidectomy with EEC reconstruction, tympanoplasty, and mastoid obliteration, enabling a more targeted surgical approach to reduce recurrence. Multiple previous middle ear surgeries were strongly associated with RP cholesteatoma (OR = 8.116, *p* = 0.010), with a 50% risk in ears with ≥ 5 prior surgeries compared with 9.2% in those with fewer. Using the association between retraction and RP cholesteatoma (OR = 4.674, *p* = 0.012) to define a risk group is not feasible preoperatively, as retraction is a postoperative finding. However, the higher occurrence of retraction in younger patients (OR = 0.175, *p* = 0.002) may help identify a subgroup at increased risk for RP cholesteatoma.

The median age for those with retraction was 11 years (IQR 8.0–23.0), compared to 25 years (IQR 12.0–40.8) for those without retraction (*p* < 0.001). When stratified by age, dry-ear rates remained high in both children ≤ 11 years and older patients, supporting the feasibility of this technique across age groups. However, younger children showed more tympanic membrane and/or posterior EEC wall retraction and a higher incidence of RP cholesteatoma, reflecting the vulnerability of the pediatric ME and Eustachian tube. This subgroup therefore requires particularly careful, prolonged clinical and MRI surveillance, with a low threshold for intervention if retraction progresses or recidivism is suspected.

The choice of graft material may also influence the development of postoperative retraction. In our series, tympanic membrane reconstruction was usually performed with temporalis fascia and/or periosteum, as many ears were revision CWD cases with little or no usable cartilage remaining. In our study, postoperative retractions of the posterior–superior EEC wall occurred in some cases years after surgery, after the reconstruction was already mature and the posterior–superior EEC wall was well formed and rigid. This illustrates how pronounced the negative pressure and retraction forces were in this population.

In selected high-risk individuals, particularly those with multiple previous COM operations due to extensive, recurrrent cholesteatoma, surgeons may consider finalizing a CWD procedure with blind-sac obliteration of the EEC and ME cleft (equivalent to subtotal petrosectomy), instead of using tympanoplasty, reconstruction of the EEC, and mastoid obliteration. Subtotal petrosectomy with blind sac obliteration of the EEC and ME cleft provides nearly 100% ear stability in the long-term [26], without requiring water precautions. Its benefits can outweigh drawbacks such as the need for surgical expertise, longer operative time, a donor site for the obliterating graft (and sometimes also a flap), and the expected significant air-conduction hearing loss. These ears typically have poor air-conduction hearing due to ossicular destruction and chronically impaired middle-ear aeration, which led to the initial development and repeated redevelopment of cholesteatoma and is unlikely to improve. Many also have a history of failed ossiculoplasty. Consequently, the likelihood of long-term ossiculoplasty success is low and hearing rehabilitation is expected to rely on bone-conduction devices. The considerations mentioned above regarding the choice of subtotal petrosectomy with blind-sac obliteration of the EEC are also true for children. Experience has been gained with children who required cochlear implantation but first needed definitive treatment for chronic otitis media or cholesteatoma in the same ear. Subtotal petrosectomy with blind-sac closure has been reported to provide a stable, dry ear with acceptable cosmetic results [27].

We compared the results of the present study with those of other studies on extensive cholesteatoma treated with CWD mastoidectomy, EEC reconstruction, tympanoplasty, and mastoid obliteration. Only studies incorporating at least 100 ears, ≥ 4 years of follow-up, and reports on key cholesteatoma outcomes, residual cholesteatoma detection methods and their application rate, as well as patient age at surgery, were included. Three relevant studies were identified (Table 4). Since outcomes in those studies were reported without considering individual follow-up durations, their table is presented as a ‘bulk’ outcome summarizing all the ears in each study. Dryness rates were similar to those obtained in our present study: 96.9%, compared to 98.5% in Gantz et al. [10]. Residual cholesteatoma rates were also comparable: 7% in this study, compared to 12.5% in Gantz et al. [10], 13.4% in Walker et al. [28], and 4.5% in Kroon [19], based on actual or estimated full application of second-look or follow-up MRI (Table 4). However, retraction was higher in the present study (33.9%) than in Gantz et al. [12] (7.6%) and Walker et al. [25] (14.0%). RP cholesteatoma was also more frequent (11.8%) compared to their studies (1.5% and 2.4%, respectively), probably due to the higher percentage of patients with previous COM surgeries in the present study (52.8% vs. 28.4% and 30%). In contrast, RP cholesteatoma rate in Kroon et al. [21] (8.0%) was closer to that in our study (11.8%), aligning with its higher rate of prior ME surgeries (54%). Kroon et al. [21] also examined ears with extensive, recurrent cholesteatoma (54% previously operated, vs 52.8% in the present study) using the same surgical technique- CWD mastoidectomy with tympanoplasty, EEC reconstruction, and S53P4 obliteration, and reported Kaplan- Meier cholesteatoma-free survival. Although their curves had a similar shape, cholesteatoma-free survival was markedly higher in their series (≈ 85% vs < 60%). This likely reflects age differences: in our cohort, 48.6% of ears were in patients < 18 years, whereas Kroon et al. included only adults (mean age 42 ± 16 vs 24.4 ± 17.6 years in our study). As shown in our Results, younger patients were at particular risk for retraction and RP cholesteatoma.

**Table 4:** Review of literature: medical outcomes of CWD mastoidectomy, EEC reconstruction, tympanoplasty, and mastoid obliteration

Year of publication, name of 1st author, ref. number	Number of ears included	Material used for Obliteration	Mean age [SD]	Percentage of ears from Patients Under 18 Years (%)	Rate of previous ME surgery	Method for res. Choles. Detection and compliance rate (%)	Achieving or developing the four outcome measures (rates calculated for the entire FU period, regardless of cases lost to FU)				
							Achieving a dry ear	Retraction development	RP choles. development	Res. choles. detection reported	Res. choles. detection based on 100% application of second look or MRI in search of res. choles
2005, Gantz [12]	130	Bone dust	31.5 [18]	31.4%	28.4%	sec. look (78%)	98.5%	7.6%	1.5%	9.8%	12.5%
2014, Walker [28]	285	Bone dust	35 [18.6]	25.6%	30%	sec look (89%)	94.4%	14%	2.4%	12%	13.4%
2022, Kroon [21]	137	S53P4 BAG	42 [16]	0%	54%	Biennial MRI (97%), sec look (3%)			8.0%	4.3%	4.5%
Luntz, present study	127	S53P4 BAG	24.4 [17.6]	49.6%	52.8%	Annual MRI (100%)	96.9%	33.9%	11.8%	7.0%	7.0%

*ref. *reference; *SD *standard deviation; *ME *middle ear, *res*. residual; *choles *cholesteatoma; *FU *follow up; *RP *retraction pocket; *sec. look *second look; *BAG *bioactive glass

### Study limitations

This study shares several limitations common to cholesteatoma research, including its retrospective design and a follow-up period that is relatively short compared with the long-time frame over which recidivism may occur. In addition, the strict inclusion criteria required to present outcomes as annual post-surgical incidences further reduced the sample size. With regard to the cholesteatoma type, the study included only ears with extensive cholesteatoma, and more than half were revision cases. Therefore, by the time of presentation for surgery, the cholesteatoma often involved both the pars tensa and the pars flaccida. This made it impossible to reliably distinguish a purely pars tensa from a purely pars flaccida origin. For this reason, and to avoid speculative classification, we did not attempt formal subclassification by cholesteatoma location/type in the analysis. In addition, postoperatively, retraction commonly involved both the reconstructed tympanic membrane and posterior–superior EAC wall as one pocket. Because the junction was often indistinct, we did not separate TM-only vs. EAC-only retraction and reported a combined variable (TM and/or posterior–superior EAC wall).

## Conclusions

In this study, CWD mastoidectomy with EEC reconstruction, tympanoplasty, and mastoid obliteration with S53P4 bioactive glass yielded high long-term rates of a dry and stable ear in extensive and recurrent cholesteatoma. Complete eradication of cholesteatoma through surgery alone remained unattainable. Younger patients and those with multiple prior surgeries were at particular risk for redevelopment of retraction and RP cholesteatoma. Cholesteatoma recidivism emerged years after surgery, which highlights the need for prolonged, structured otoscopic and MRI follow-up after surgery. Reporting annual incidence together with follow-up completeness may improve comparability between cholesteatoma outcome studies. Despite efforts to instruct patients and parents on the importance of regular long-term follow-up, attendance at follow-up visits continued to decline over time.

## Appendix

**Table 5:** Percent and number of patients who achieved/developed each one of the main outcomes out of the patients who attended the yearly FU visits: for the entire group of participants, for patients ≤11 and for patients >11 years

	All patients	≤11 years	>11 years	*P*-value*
% Achieving a completely epithelized and dry ear (n/N)				
1y	92.1 (117/127)	93.9 (31/33)	(86/94) 91.5	0.697
2y	89.6 (95/106)	82.8 (24/29)	92.2 (71/77)	0.043*
3y	82.9 (63/76)	80.0 (16/20)	83.9 (47/56)	0.356
4y	85.4 (35/41)	80.0 (8/10)	87.1 (27/31)	0.348
% Retraction of the TM and or the posterior EEC wall development (n/N)				
1y	13.4 (17/127)	27.3 (9/33)	8.5 (8/94)	0.014*
2y	26.4 (28/106)	37.9 (11/29)	22.1 (17/77)	0.137
3y	28.9 (22/76)	45.0 (9/20)	23.2 (13/56)	0.087
4y	34.1 (14/41)	50 (5/10)	29.0 (9/31)	0.267
% RP cholesteatoma development (n/N)				
1y	0.8 (1/127)	0 (0/33)	1.1 (1/94)	>0.999
2y	4.7 (5/106)	13.8 (4/29)	1.3 (1/77)	0.019*
3y	9.2 (7/76)	20.0 (4/20)	5.4 (3/56)	0.073
4y	4.9 (2/41)	0 (0/10)	6.5 (2/31)	>0.999
% Residual cholesteatoma detection on Non-EP DW MRI (n/N)				
1y	0.8 (1/127)	0 (0/33)	1.1 (1/94)	>0.999
2y	2.8 (3/106)	3.4 (1/29)	2.6 (2/77)	>0.999
3y	3.9 (3/76)	0 (0/20)	5.4 (3/56)	0.562
4y	4.9 (2/41)	20.0 (2/10)	0 (0/31)	0.055

*p*-value refers to the comparison between the two age groupes.**P*-value < 0.05

*FU* follow up; *n* number of patients who achieved/developed each one of the main outcomes; *N* number of patients who attended the yearly FU visit; *TM* tympanic membrane; *EEC* external ear canal; *RP* retraction pocket; *Non-EP DW MRI* non-eco-planar diffusion-weighted magnetic resonance imaging
